# Evidence from district level inputs to improve quality of care for maternal and newborn health: interventions and findings

**DOI:** 10.1186/1742-4755-11-S2-S3

**Published:** 2014-09-04

**Authors:** Rehana A Salam, Zohra S Lassi, Jai K Das, Zulfiqar A Bhutta

**Affiliations:** 1 Division of Women & Child Health, Aga Khan University, Karachi, Pakistan; 2 Program for Global Pediatric Research, Hospital For Sick Children, Toronto

**Keywords:** Quality of care, district, maternal health, newborn health, governance, accountability, supervision, financial incentives, information system

## Abstract

District level healthcare serves as a nexus between community and district level facilities. Inputs at the district level can be broadly divided into governance and accountability mechanisms; leadership and supervision; financial platforms; and information systems. This paper aims to evaluate the effectivness of district level inputs for imporving maternal and newborn health. We considered all available systematic reviews published before May 2013 on the pre-defined district level interventions and included 47 systematic reviews.

Evidence suggests that supervision positively influenced provider’s practice, knowledge and client/provider satisfaction. Involving local opinion leaders to promote evidence-based practice improved compliance to the desired practice. Audit and feedback mechanisms and tele-medicine were found to be associated with improved immunization rates and mammogram uptake. User-directed financial schemes including maternal vouchers, user fee exemption and community based health insurance showed significant impact on maternal health service utilization with voucher schemes showing the most significant positive impact across all range of outcomes including antenatal care, skilled birth attendant, institutional delivery, complicated delivery and postnatal care. We found insufficient evidence to support or refute the use of electronic health record systems and telemedicine technology to improve maternal and newborn health specific outcomes.

There is dearth of evidence on the effectiveness of district level inputs to improve maternal newborn health outcomes. Future studies should evaluate the impact of supervision and monitoring; electronic health record and tele-communication interventions in low-middle-income countries.

## Introduction

District level healthcare is the cornerstone of primary health. An ideal district health system should not only offer primary care services but also provide first level of outpatient care and referrals for more specialized care. They also serve as a nexus between community and facility level care for health information; play a direct role in training health care workers; and provide necessary data to guide national health policy. This role is fundamental to effective health care delivery and failure to recognize the interrelationship between community and district-level facilities might result in inefficiency and fragmented delivery of meaningful public health interventions. Community based intervention impacts discussed in paper 2 of this series [1] could not be achieved if district level priorities do not reflect the needs of the community.

District level facilities play a pivotal role for maternal newborn health (MNH) programs. In some countries, programs like Safe Motherhood Initiative and Integrated Management of Childhood Illness (IMCI) are based on district-level health systems. Outpatient clinics at district hospitals provide primary prevention services for MNH including universal maternal and childhood immunizations. However these programs may vary in structure and functioning from country to country depending on the healthcare needs and infrastructure. The core components of district level inputs include training, supervision and monitoring of health workers in the peripheral health centers and managing health information systems for strategic planning and monitoring of the district health system. In this paper, we have reviewed the effectiveness of care delivered through district level inputs for improving MNH outcomes. For this review we have broadly categorized these interventions into four categories: governance and accountability mechanisms; leadership and supervision; financial incentives; and information systems.

## District level characteristics

### Governance and accountability mechanisms

Governance is achieved through a combination of strategies including clinical competence, patient involvement, risk management, use of information, staff management, maintaining medical registries, and implementation of continuous quality improvement (CQI) tools. Accountability involves audit and feedback mechanisms that entail a systematic approach to ensure that the services are accountable for delivering quality healthcare. Audits involve any summary of clinical performance of healthcare professionals over a period of time which is presented to them in a written, electronic or verbal format for self-accountability. Healthcare professionals are prompted to modify their practice if the feedback is inconsistent with the standards or accepted guidelines. Audit tools for evaluating maternal and perinatal deaths have been an integral part of quality improvement in obstetric care. These are effective in defining the context specific problem and propose solutions. Although these mechanisms have been used widely as a strategy to improve professional practice, they have not shown consistent effectiveness majorly due to the inconsistencies and variations involved in implementation [2,3].

### Leadership and supervision

Supervision plays a key role in primary healthcare (PHC) service delivery and it requires the district level staff to supervise the public health activities and provide appropriate clinical care [4-6]. Good leadership is also critical to the success of district health systems as it relies on how leaders work together to enable the health system to achieve its goals [7]. It involves strategic planning for the provision of services, resource allocation, and set priorities for improved performance. Aspects of leadership and supervision involve problem solving, reviewing records, observing clinical practice, mentoring and guidance on matters of personal, professional and educational development in context to patient care. Supervision in district health structures is difficult to implement due to time and costs involved and also the increasing numbers of district level facilities in even increasingly remote areas [8]. One of the emerging concepts is the involvement of local opinion leaders in district health leadership to promote knowledge transfer of evidence into practice and ultimately improve health care [9]. Since these individuals are perceived as credible and trustworthy, they may play a key role in assisting individuals to identify the best evidence-based healthcare practice and facilitate behavior change [10].

### Financial incentives

It involves provision of monetary benefits as a source of motivation for performing desired health related actions. Financial interventions are aimed at creating a greater demand for health services and include scale-up of preventive health interventions, as well as provision of free access to basic health care. In recent years there has been an increase in the utilization of financial support platforms to reduce out of pocket client expenditure and strengthen service delivery and utilization at the district level. The targeted health services can include seeking care, behavior modification, immunization, compliance to health professional’s clinical behavior and performance. Incentives directed towards care providers include capitation (payment for each patient enrolled), fee for service and pay for performance. Those directed towards users involve conditional cash transfers (CCT), vouchers, health insurance and fee exemptions. Diverse and innovative financial support platforms are being implemented in some of the fragile states such as Cambodia, Afghanistan, Pakistan and Haiti as well as more established economies of Latin American countries [11,12] however, impact on quality of care for MNH is still emerging [13].

### Information system

It is one of the essential building blocks of health system [14] that captures, manages and transmits information related to the health of individuals or activities of organizations. It involves district level routine information systems, disease surveillance, hospital patient administration, electronic health records, human resource management and communication systems. Health information system is vital for public health decision making, health sector reviews, planning and resource allocation and program monitoring and evaluation. Weak information systems are a critical challenge to achieving the MNH related Millennium Development Goals. The major challenges identified in this domain include issues related to completeness, accuracy and timeliness, especially in low middle income countries (LMIC). These challenges limit its use in routine district health care planning, monitoring, and evaluation. Other factors associated with poor quality data in resource constrained settings include duplicate, parallel reporting channels and insufficient capacity to analyze and use data for decision making. Recently, there is an increased emphasis on utilizing electronic communication systems including mobile phones, telephone based follow-up and counseling, after-hours telephone access, and screening. As the field is still nascent, a limited but growing body of evidence exists to support the role of mobile technologies in improving MNH outcomes [15-17]. Despite the anticipated benefits of mHealth; wide-scale impacts of mHealth on MNH outcomes need to be explored further [18,19].

We aim to systematically review and summarize the available evidence from relevant systematic reviews on the impact of the outlined district level inputs (panel 1) to improve the quality of care for women and newborns.

**Table 1:** Components of district level interventions

**Governance and accountability:** Any systematic approach to ensure that services are accountable for delivering quality healthcare including audit and feedback mechanisms, medical registries, and continuous quality improvement tools. **Leadership and supervision:** Supervision is provision of monitoring, guidance and feedback on matters of personal, professional and educational development in the context of the patient care. Good leadership involves strategic planning for the provision of services, resource allocation, and set priorities for improved performance. **Financial strategy:** It involves provision of monetary benefits as a source of motivation for performing desired health related actions. Financial incentives can be user- as well as provider-directed. **Information systems:** It refers to a system that captures, manages and transmits information related to the health of individuals or the activities of organizations. Two emerging components of the information systems are the electronic health records and electronic communications. Electronic health record is the provision and access to electronically retrievable health records at the point of healthcare delivery while electronic communication involves computerized communication, telephone follow-up and counseling, interactive telephone systems, after-hours telephone access, and telephone screening.

## Methods

We considered all available systematic reviews on the pre-defined district level interventions published before May 2013 as outlined in our conceptual framework [20]. A separate search strategy was developed for each component using pre-identified broad keywords, medical subject heading (MeSH), and free text terms: [(Governance OR accountability OR audit OR feedback OR leadership OR supervis* OR financ* OR incentive OR “cash transfer*” OR CCT OR voucher OR insurance OR “user fee*” OR exemption OR “pay for performance” OR record* OR data OR electronic “electronic data” OR “information system” OR “electronic information system” OR “electronic communication” OR “telecommunication” OR mhealth OR ehealth) AND (health OR healthcare OR maternal OR mother OR child OR newborn OR “neonat*”)]

Our priority was to select existing systematic reviews, which fully or partly address the *a priori* defined district level interventions for improving quality of care for MNH. We excluded the reviews pertaining to nursing documentation, computerized pharmacy system or those focusing on certain specific chronic illnesses only as these were not included in the scope of our review. Search was conducted in the Cochrane library and PubMed and reviews that met the inclusion criteria were selected and data was abstracted by two authors on a standardized abstraction sheet: Quality assessment of the included reviews was done using Assessment of Multiple Systematic Reviews (AMSTAR) criteria [21] as detailed in paper 2 of the series [20]. Any disagreements between the primary abstractors were resolved by the third author. For the pre-identified interventions, which did not specifically report MNH outcomes, we have reported the impacts on other health outcomes as reported by the review authors. Estimates are reported as relative risks (RR), risk ratios (RR), risk differences (RD) or mean differences (MD) with 95% confidence intervals (CI’s) where available. For detailed methodology please refer to paper 1 of the series [20].

## Findings

**Figure 1:**
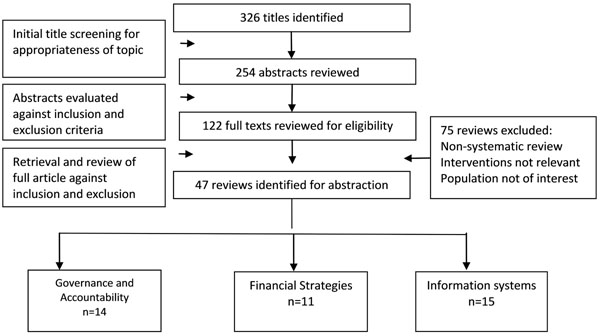
Search flow diagram

Our search identified 326 potentially relevant review titles. Further evaluation of the abstracts and full texts resulted in the inclusion of 47 eligible reviews: 14 on governance and accountability mechanisms, 7 on leadership and supervision, 11 on financial strategies and 15 on information systems (Figure 1). The overall quality of reviews ranged from 3 to 10 on the AMSTAR criteria with a median score of 8.

### Governance and accountability

We included 14 [22-35] reviews evaluating the effectiveness of governance and accountability mechanisms. The median quality score was 7.5 on AMSTAR rating scale. Most of the reviews evaluated a set of pre-selected process and outcome indicators as the outcomes reported in the individual studies varied widely. Three reviews reported MNH related outcomes [22,26,29] including immunization rates, mammography uptake, perinatal and maternal morbidity and mortality. Other reported outcomes included compliance, performance improvement and rate of prescription for generic drugs. Most of the studies included in these reviews were conducted in high income countries (HIC). The characteristics and findings of the included reviews are presented in Table 2.

**Table 2:** Characteristics of the reviews included for governance and accountability

Reviews (n=14)	Description of included interventions	Type of studies included (number)	Targeted health care providers	Outcome reported		Pooled data (Y/N)	Results
						
				Other outcomes	MNH specific outcomes		
**Bordley 2000 **[22]	Audit and feedback was defined as any summary of clinical performance gathered over a defined period of time and presented to the health care provider after collection.	ITS: 6 RCT: 5 Pre-post: 4	Health care professionals		Immunization rate	No	17% absolute decrease to 49% increase

**Grimshaw 2004 **[23]	Audit and feedback: any summary of clinical performance of healthcare over a specified period.	C-RCTs: 110 P-RCTs: 29 C-CCTs: 7 PCCT: 10 CBAs: 40 ITS: 39	Health care professionals	Performance improvement		No	Absolute improvement +7.0% (range +1.3 to +16.0%) (dichot process measures)

**Hulschur 2001 **[24]	Feedback: provision of a summary of clinical performance after the performance concerned, based on medical records, computerized data-bases or other sources of information.	55 studies: RCTs:37 nRCTs:18	Primary care professionals directly accessible to patients for all types of health problems in US	Preventive services		No	Absolute increase of 3% to 26%
							0.8 more visits

**Ivers 2012 **[35]	Audit and feedback defined as any summary of clinical performance over a specified period of time	RCT: 49	Health care provider (excluding students)	Compliance		Yes	4.3% absolute increase in healthcare professionals’compliance with desired practice (dichot)
							1.3% absolute increase in healthcare professionals’compliance with desired practice (cont)

**Jamtvedt 2006 **[25]	Audit and feedback defined as any summary of clinical performance over a specified period of time	RCT: 118	Health care provider (excluding students)	Compliance		No	median-adjusted risk difference was 5% (range 3–11) (dichot)
							median-adjusted percentage change relative to control was 16% (5–37)

**Jepson 2000 **[26]	Audit and feedback to physicians on their performance, and sometimes that of their peers	05 studies: RCTs: 02 quasi-RCT:01 Controlled trials: 02	All people eligible to participate in a screening programs as defined by the entry criteria for that programs, included population groups such as pregnant women, neonates, children and adults in US		Screening Uptake	No	One trial: no effect on screening for occult blood
							One trial and one quasi: feedback more effective on some tests
							Two trials: increased uptake of mammograms (p<0.05)

**Johnston 2000 **[27]	Clinical and Medical Audit mechanisms	Total Studies: 93	All health professionals, mostly in UK	Clinician's perceptions of benefits and disadvantages of audit.		No	Narrative
				Barriers and facilitators of audits.			

**Oxman 1995 **[28]	Audit and feedback: Any summary of clinical performance of health care over a specified period, with or without recommendations for clinical action.	Total: 31	Health care provider (excluding students) in mixed country setting	Rate of prescription for generic drugs		No	40% increase in rate of prescription

**Pattinson 2005 **[29]	Any form of audit and feedback with any other clearly defined form of audit or feedback or control group	No studies	Maternity units	Time and costs	Perinatal and maternal mortality and morbidity rates	No	No studies found
				Conflicts			

**Phillips 2010 **[30]	Clinical governance is a systematic and integrated approach for ensuring services is accountable for delivering quality health care. Clinical governance is delivered through a combination of strategies including: ensuring clinical competence, clinical audit, patient involvement, education and training, risk management, use of information, and staff management.	RCTs: 7, longitudinal observational: 11 Case study: 1	Primary health care providers in HIC	Process measures		No	Narrative
				Outcome measures			

**Pyone 2012 **[31]	Not clearly defined	Total: 2	Staff, obstetricians and community		Maternal mortality and CFR	No	Narrative

**Scott 2009 **[32]	Clinical governance defined as Systematic coordination and promotion of activities that contribute to continuous improvement of quality of care: clinical audit; clinical risk management; patient/service user involvement; professional education and development; clinical effectiveness research and development; staff focus; use of information systems; and institutional clinical governance committees. Separate definition of audit and feedback not given.	Total: 118	General Physicians, mostly in HIC	Compliance		No	Median increase in compliance 5% (dichot) and 16% for continuous
				Patient health outcomes			

**Veer 2010 **[33]	Medical registry defined as a systematic and continuous collection of a defined data set for patients with specific health characteristics.	Studies:53	Health Care Professionals	Process measures		No	26 of 43 process measures were positively influenced
				Outcome measures			5 of 36 outcome measures were positively influenced

**Wensing 1998 **[34]	Any interventions influencing the implementation of guidelines and adoption of innovations in general practice. Feedback not defined.	Total: 143 RCTs: 39, CBA: 22, nRCTs: 13 non randomized, uncontrolled trials: 67	GPs in HIC	Guideline implementation and adoption of innovations		No	Effective in 10 of 15 groups

The effectiveness of audit and feedback mechanisms varied widely for various outcomes ranging from nil to moderate effect. Implementation involved a baseline audit followed by rounds of audit and feedback at defined intervals. In some settings audit and feedback was provided in combination with financial incentives. Audit and feedback was found to be positively associated with childhood immunization with the effect estimate ranging from 17% absolute decrease to 49% increase. However, the exact magnitude could not be ascertained due to the limited number of low quality design studies [22]. For the screening uptake, feedback resulted in higher proportion of physicians with completed mammograms and it was most effective when targeting test ordering and prevention activities, and when associated with low baseline adherence to recommended care or more intense feedback [26]. A review evaluating the effectiveness of maternity ward audits did not find any trial for inclusion but reported that serial data suggests benefit [29].

For outcomes other than MNH, audit and feedback was found to improve health care workers performance and compliance with desired practice by 7% and 4.3% respectively [23,35]. It was also associated with 40% increase in rate of prescription for generic drugs [28]. Feedback involved verbal, written or both provided either by the supervisor, professional standards review organization or employer representative. Majority of the feedback provided included action plans or correct solution information with the feedback. Some of the reported factors influencing audit and feedback included problems with staff coordination, lack of strong evidence base for some topics, poor access to published work and high-quality clinical data, organizational factors and lack of time and motivation [32,33].

### Leadership and supervision

We included seven [28,36-41] reviews evaluating the impact of leadership and supervision with a median quality score of 8 on AMSTAR criteria. Included reviews focused on the impact of leadership and supervision for the primary health workers [36,37]; involvement of local community leaders [38], nursing leadership [39,41] and supervising counselors or psychotherapist [40]. Due to the wide range of reported outcomes, data could not be pooled for any outcome except for compliance in one review evaluating the impact of involving opinion leaders [38]. None of the reviews reported outcomes specific to MNH while other reported outcomes included compliance, patient satisfaction, provider’s practice and knowledge. The evidence was from both LMIC and HIC. The characteristics and findings of the included reviews are presented in Table 3.

**Table 3:** Characteristics of the reviews included for leadership and supervision

Reviews (n=07)	Description of included interventions	Type of studies included (no)	Targeted health care providers	Outcome reported		Pooled data (Y/N)	Results
						
				Other outcomes	MNH specific outcomes		
**Capblanch 2008 **[36]	Summarize opinion about what supervision of primary health care is by those advocating it; compare these features with reports describing supervision in practice; appraise the evidence of the effects of sector performance.	Total: 74 Policy and opinion papers: 08 Descriptive Studies: 54 Quasi: 12	PHC Workers in LMIC	Health service coverage		No	10 of 11 studies showed at least one outcome favoring intervention.
				Knowledge and awareness			

**Capblanch 2011 **[37]	Supervision is conceptualized as the link between district and peripheral health staff, and is considered important in staff motivation and performance. Supervision often includes aspects of problem solving, reviewing records and observing clinical practice. Supervision mostly means visiting supervisees, but also includes meetings in the centre.	Total: 09 cRCT’s: 05 CBA: 04	PHC Worker in LMIC	Providers’ practice		No	2 of 3 studies found positive impact
				Providers’ knowledge			1 of 3 studies found positive impact

**Flodgren 2011 **[38]	Opinion leaders had to be identified by one of the following methods: socio-metric method, informant method, self-designating method, observation method.	RCT’s: 18	Local opinion leaders in HIC	Compliance		Yes	RD12% (6- 14.5%)

**Oxman 1995 **[28]	Use of providers explicitly nominated by their colleagues to be "educationally influential”.	Trials: 04	Local opinion leaders		No of vaginal deliveries	No	Increase in number of vaginal deliveries after C-section in hospitals where local opinion leaders were involved (1/1)

**Pearson 2007 **[39]	Feasibility, meaningfulness and effectiveness of developing and sustaining nursing leadership to foster a healthy work environment in healthcare.	Total:48 Review: 1 Experimental: 2 Descriptive correlational: 45	Nursing Personnel	Satisfaction		No	Narrative

**Wong 2007 **[41]	Leadership was defined as the process through which an individual attempts to intentionally influence another individual or a group to accomplish a goal.	Observational: 07	Nursing Personnel	Patient satisfaction		No	Satisfaction increased in 2 of 3 (insignificant in 1)
				Patient mortality and patient safety			Mortality reduced in 1 of 3 (insignificant in 2)
				Adverse events			Adverse events decreased in 2 of 3 (insignificant in 1)
				Complications			Complications decreased in 2 of 3 (insignificant in 1)

**Wheeler 2007 **[40]	Supervisors must be counselors or psychotherapists or other professionals who have had a substantial training as counselors or psychotherapists and who were specifically engaged in a counseling role with clients.	Quantitative: 08 Qualitative: 03 Mixed: 07	Counselors or psychotherapist in HIC	Self-awareness, skills, self-efficacy, timing and frequency, theoretical orientation, support , client outcomes		No	Narrative

Involving local opinion leaders to promote evidence-based practice resulted in a 12% [RD: 12%, 95% CI: 6- 14.5%] absolute increase in compliance with the desired practice [38]. These opinion leaders were identified using the sociometric method in which healthcare professionals were asked to complete a self-administered questionnaire to identify educationally influential colleagues. Once identified, they were involved in informal or formal teaching through one to one teaching, community outreach education visits, small group teaching, preceptor-ships and delivering. Involving local opinion leaders was found comparable to other strategies used to disseminate and implement evidence based practice in health care including distribution of educational materials, audit and feedback and educational outreach. Another review based on a single RCT demonstrated a substantial increase in the number of trials of vaginal delivery after previous cesarean section in hospitals with the involvement of local opinion leaders [28]. The impact of supervision on the quality of primary health care in LMIC was inconclusive due to low quality studies [37]. Nursing leadership and supervision suggested improvements in patient satisfaction and reduction of adverse events; however, the evidence is inconclusive for complications and mortality rates [39,41].

### Financial strategy

We included 10 [12,42-50] reviews and 1 [11] overview of reviews with median data quality score of 8.5 on AMSTAR criteria. Six reviews evaluated provider-directed incentives in the form of pay for performance, economic incentives, results based financing (RBF), salary, capitation or fee-for-service (FFS); while others focused on user-directed incentives including CCTs, vouchers, health insurance or user fee exemption. Seven reviews reported outcomes specific to MNH while meta-analysis was conducted in only two of the reviews [42,50]. Reported MNH outcomes included immunization coverage, service utilization, institutional delivery, antenatal care (ANC), post natal care (PNC), skilled birth attendant, child nutritional status and anthropometry while other reported outcomes were consultation rates, compliance, prescription rates, referrals and hospital/Emergency Department (ED) visits. Most of the reviews were from LMIC. The characteristics and findings of the included reviews are presented in Table 4.

**Table 4:** Characteristics of the reviews included for Financial Platforms

Reviews (n=11)	Description of included interventions	Type of studies included (no)	Targeted health care providers	Outcome reported*		Pooled data (Y/N)	Results
						
				Other outcomes	MNH specific outcomes		
**Flodgren 2011 **[11]**(overview)**	An incentive is any factor (financial or non-financial) that provides motivation for a particular course of action, or counts as a reason for preferring one choice compared to alternatives. Financial incentives are extrinsic sources of motivation and exist when an individual receives a monetary transfer which is made conditional on acting in a particular way	4 reviews	physicians, dentists, nurses, and allied healthcare professions (such as physiotherapists, speech therapists etc.) involved in providing direct patient care in LMIC and HIC	Consultation or visit rates		No	Improvement in 10/17 outcomes
				Processes of care			Improvement in 41/57 outcomes
				Referrals and admissions			Improvement in 11/16 outcomes
				Compliance			Improvement in 5/17 outcomes
				Prescribing costs			Improvement in 28/34 outcomes

**Gaarder 2010 **[42]	The traditional CCT programs (which is how we will refer to the nine safety-net type of programs included in the study) were specifically designed to influence demand-side factors, and, in most cases, not the supply-side factors	41 studies related to 11 programs/interventions	General population		Clinic visits	Yes	1.26 (1.09, 1.45)
					Immunization-DPT		1.08 (1.03, 1.14)
					Immunization-Full		1.09 (0.97, 1.22)
					Nutritional improvements-stunting		1.04 (0.92, 1.18)
					Nutritional improvements-wasting		1.19 (0.55, 2.57)

**Giuffrida 1999 **[43]	Target payments remuneration. Under a target payments remuneration system a lump sum payment is made if, and only if, the PCP reaches a predetermined quantity or target level of care.	RCT: 1 ITS: 1	Primary Care Physicians (PCPs) defined as medically qualified physicians who provide primary health care.		Immunization rates	No	Significant improvement in 1 of 2 studies

**Gosden 2000 **[44]	**Salary:** where a lump sum payment is made to the PCP for a set number of working hours or sessions per week. **Capitation**: where a payment is made to a PCP for every patient for whom they provide care. **Fee-for-service (FFS):** where payment is made to a PCP for every item of service or unit of care that they provide.	RCTs: 2, BFA: 2	Primary Care Physicians in HIC	Primary care physician visit		No	Narrative
				Prescriptions			
				Diagnostic and curative services			
				Referrals			
				Health/emergency department visits			
				Hospitalization			
				Compliance			
				Costs			

**Lagarde 2007 **[12]	effect of directly transferring money to households conditional on some requirements, at least 1 of which had to be related to health seeking behavior	Total: 10 RCTs:04, quasi-randomized trial: 01 controlled before-and-after study: 1	People living in low- or middle-income countries, as defined by the World Bank. Health services and institutions in LMIC	Care seeking behavior	Immunization coverage	No	5/5 studies showed significant improvement in at least 1of the care seeking outcome
					Anthropometric and nutritional		3/4 studies reported significant improvement All programs showed positive outcome

**Lagarde 2009 **[45]	Direct monetary transfers made to households and transfers conditioned on a particular behavior or action (e.g. visit to a health facility for regular checkups). Unconditional transfers were not considered.	RCT’s : 08, controlled before after (CBA) studies: 02	People living in low- or middle-income countries, as defined by the World Bank. Health services and institutions		Health service utilization	No	27% increase in individuals returning for voluntary HIV counseling, 2.1 more visits per day to health facilities
					Immunization coverage		11-20% more children taken to the health center 23-33% more children<4 yrs. attending preventive healthcare visits 3/4 showed improvement (insignificant)
					Health outcomes		22-25% decrease in the probability of children <3 years old being reported ill in the past month
					Child anthropometry		3/4 studies reported improvement (1 negative)

**Oxman 2009 **[46]	RBF can be defined as the transfer of money or material goods conditional on taking a measurable action or achieving a predetermined performance target		recipients of healthcare, individual providers of healthcare, healthcare facilities, private sector organizations, public sector organizations, sub-national governments (municipalities or provinces), national governments, or multiple levels in LMIC	TB outcomes		No	Narrative
				Program specific outcomes			

**Scott 2011 **[47]	Financial incentives defined in detail in terms of method of payment, level of payment. Quality of care defined broadly as of “the degree to which health care services for individuals and populations increase the likelihood of desired outcomes and are consistent with current professional knowledge	cRCT: 3CBA:2 ITS: 1	Primary care physicians (PCPs): PCPs are defined as doctors holding A medical degree and include general practitioners, family doctors, family physicians, family practitioners, and other generalist physicians working in primary healthcare settings who fulfill primary health care tasks	Quality of care			6/7 studies showed modest positive effects on quality of care for some primary outcome measures, but not all. One study found no effect on quality of care

**Town 2005 **[48]	The term “economic incentives” describes financial incentives where there is an increase in physician income that is a function of measurable performance criteria. These include bonus payments payable on the basis of number of specific services provided, or based on the provider achieving a target outcome or target behavior.	RCT’s: 06	Physician in US		Preventive services	No	1/6 studies reported significant improvement

**Witter 2012 **[49]	Pay for performance refers to the transfer of money or material goods conditional on taking a measurable action or achieving a predetermined performance target	RCT: 1, CBA: 6, interrupted time series: 2	providers of healthcare services (health workers and facilities), sub-national organizations (health administrations, non-governmental organizations or local governments), national governments and combinations of these in LMIC		Provider performance (QoC)	No	Mixed findings from 5 studies Both positive and negative impacts in 2 studies
					Utilization of service (antenatal care)		Mixed findings from 4 studies
					Utilization of service (institutional delivery)		No impact on preventive care
					Utilization of service (preventive care for children)		Immunization coverage improved in 4/4 studies
					Patient outcome		Improved wasting in 1/1 study

**Zaidi 12 (unpublished)**[50]	Financing platforms that addressed maternal care either as primary objective of their study or as part of a larger service package. Types of financing strategies considered for this review included cash transfers, vouchers, contracting, community health insurance schemes, national health insurance, and user fee exemption.	12	General population		**Maternal Voucher Schemes:**	Yes	**2.97 (2.38-3.71)**
					Institutional delivery		3.70 (2.03-6.73)
					Skilled birth attendant		3.81 (2.92-4.95)
					Complicated delivery		1.53 (1.14-2.05)
					ANC		3.08 (2.23-4.25)
					PNC		2.66 (1.59-4.44)
					**Maternal CCT:**		**0.88 (0.76-1.02)**
					Skilled birth attendant		0.88 (0.76-1.02)
					**User fee removal:**		**1.57 (1.33-1.85)**
					Institutional delivery		1.58 (1.16-2.14)
					Skilled birth attendant		1.54 (1.26-1.88)
					**National health insurance:**		**1.22 (0.90-1.65)**
					ANC		1.04 (1.01-1.07)
					Institutional delivery		1.48 (0.79-2.78)
					**Community based health insurance:**		**1.77 (1.29-2.44)**
					Institutional delivery		3.00 (1.60-5.61)
					ANC		1.41 (1.22-1.63)
					PNC		0.96 (0.46-2.00)

Among user directed financial strategies, CCT demonstrated significant improvements in preventive clinic visits (RR: 1.26, 95% CI: 1.09, 1.45), Diphtheria, pertussis and tetanus (DPT) immunization (RR: 1.08, 95% CI: 1.03, 1.14), health service utilization, child nutritional status and health outcomes [42,45] with non-significant impacts on full immunization, stunting and wasting [42]. An unpublished review on the impact of a range of financial platforms on MNH reported significant overall impact on maternal health indicators with maternal voucher schemes (RR: 2.97, 95% CI:2.38-3.71), user fee exemption (RR: 1.57, 95% CI: 1.33-1.85) and community based health insurance (RR: 1.77, 95% CI: 1.29-2.44) while CCTs and national health insurance (NHI) did not show any significant impacts [50]. Maternal voucher schemes were reported to be the most effective strategy and demonstrated significant improvements across all range of outcomes including institutional delivery (RR: 3.7, 95% CI: 2.03, 6.73), skilled birth attendance (RR: 3.81, 95% CI: 2.92, 4.95), complicated delivery (RR: 1.53, 95% CI: 1.14, 2.05), ANC (RR: 3.08, 95% CI: 2.23, 4.25) and PNC (RR: 2.66, 95% CI: 1.59, 4.44) [50].

Among provider-directed financial strategies, target payments to primary care physicians (PCP) and pay-for performance showed positive trends for immunization rates [43] while the findings were inconclusive for provider performance, service utilization, compliance or quality of primary health care [11,43,47,49]. We did not find any evidence for the impact on patient outcomes.

### Information systems

We included fifteen [19,51-65] reviews pertaining to computerized communication, electronic health record system, telephone follow-up and counseling, interactive telephone systems, after-hours telephone access and telephone screening. The quality of included reviews ranged from 3 to 10 on AMSTAR criteria. Reported MNH outcomes included immunization rates, mammography uptake, and newborn health outcomes [51,64] while other reported outcomes included technology adoption, patient satisfaction, professional behavior and knowledge. All the reviews were from HIC only. The characteristics and findings of the included reviews are presented in Table 5.

**Table 5:** Characteristics of the reviews included for Information System

Reviews (n=15)	Description of included interventions	Type of Studies included (no)	Targeted Health care Providers	Outcome reported		Pooled Data (Y/N)	Results
						
				Other outcomes	MNH specific outcomes		
**Black 2011 **[52]	The researchers divided e-Health technologies into three main categories: (1) storing, managing, and transmission of data; (2) clinical decision support; and (3) facilitating care from a distance.	53 systematic reviews	Various health care professionals	Patient outcomes	Electronic prescribing	No	Weak to moderate effect (10 out of a total of 26 studies)
					Aassociated computerised provider (or physician) order entry systems		6 out of a total of 6 studies showed no benefit

**Gagnon 09 **[65]	Any type of intervention to promote the adoption and use of any type of Information Communication Technology (ICT) (electronic medical record, telemedicine/ tele-health, health information networks, decision support tools, Internet-based technologies and services).	RCT: 09 ITS: 01	healthcare professionals, residents, fellows, and other registered healthcare professionals in HIC	Information and communication technology adoption		No	Small to moderate positive effect on adoption (4/10)
							No significant positive effect (4/10)
							Mixed effect (2/10)

**Hayrinen 08 **[58]	Electronic health records: definitions, structure, context, access, purpose and methods	89 papers	Health care professionals in HIC	Electronic health records: definitions, structure, context, access, purpose and methods		No	Narrative

**Irani 09 **[60]	Use of Electronic health record system in outpatient and office setting	Cross-sectional: 03 Pre-post: 04 (meta-analyzed: 03)	Physicians in HIC	Patient satisfaction		Yes	3.7% (2.9-5.2%)

**McGowan 09**[61]	Provision and access to electronically retrievable health records at point of healthcare delivery and training component	cRCT’s: 02	Physicians, nurses and midwives in HIC	Professional behavior		No	No significant change (2/2)
				Improvement in knowledge			Improved knowledge (1/2)

**Ballas 1997 (electronic communication)**[51]	**Distance technology applications were described in 6 categories: computerized communication, telephone follow-up and counseling, telephone reminders, interactive telephone systems, after-hours telephone access, and telephone screening.**	**80 clinical trials**	**General healthcare providers**	Computerized communication: **Clinical outcomes for diabetes, Alzheimer’s and cardiac diseases**		**No**	**HbA_1c_ decreased (4/4)** **Insignificant changes in other outcomes**
				Telephonic follow-up: **ED visits**			**Significant improvements in keeping appointment, compliance, follow-up care, satisfaction**
				**Cardiac care**			**Significant improvement in smoking cessation, exercise, general activity, knowledge**
					**Mammography use**		**Significant increase in no of mammograms (Range: 14%-25%)**
				**Osteoarthritis**			**Significant improvement in AIMS score**
				**Tobacco use prevention**			**Significant decrease of 8.3%**
				**Appointment keeping rates**			**Appointment kept or responsibly cancelled (5/6)**
					**Immunization rates**		**Significant increase (Range: 6.4%-27.2%)**
				**Medication compliance**			**Significant improvement in compliance events and pharmacy score**
				**Diabetic foot care**			**Significant improvement in Serious foot lesions, dry or cracked skin, ingrown toenails and fungal nail infections**
				**Osteoarthritis**			**Significant improvement in physical disability and pain**

**Bunn 04/ Bunn 05 (tele consultation) **[53,54]	All designated telephone consultation systems where patients calls are received, assessed and managed by giving advice or by referral to a more appropriate service. This included those with and without computer based clinical decision support systems	RCT: 05 CCT:01 ITS: 03	healthcare providers in HIC	Visit to GP’s		No	Significant reduction (3/5)
				Visits to A&E department			No difference (6/7), significant increase (1/7)
				Hospital admissions			Reduction in hospital admissions (2/2)
				Home visit			No significant reduction (1/1)
				Out of hours contact			Small significant increase (1/2)
				Patient satisfaction			
				Cost			

**Car 11 **[55]	Online health literacy	RCT: 01 CBA: 01	All patients/ consumers	Self-efficacy for health information seeking		No	1.10 points higher in intervention group
				Health information evaluation skills			0.60 points higher in intervention group
				# of times pt. discussed online			0.7 times higher in intervention group

**Currell 00 (telemedicine) **[56]	Studies which compare the provision of patient care face to face with care given using telecommunications technologies, in which at least two communication media are used interactively (e.g. video consultation between hospital consultant and general practitioner).	Trials: 07	Qualified healthcare practitioners from any discipline in HIC	Measurable difference in outcome of care		No	No unequivocal benefit (7/7)
				Economic consequence			
				Acceptability of care			
				Difference in professional practice			
				Difference in transfer of care			

**Grilli 02**	Mass media	ITS: 20	Healthcare providers patients and general public	Effectiveness		No	Effective in improving healthcare utilization (7/7 studies)

**Heselmans 09 **[59]	An electronic guideline implementation method was defined as an electronic system directly supporting evidence-based clinical decision making in which point-of care advice is provided based on one or more CPGs	20 cRCT, 1 CCT, 2 CBA	physicians	Patient outcomes		No	7/23 studies had >50% of the process outcomes significantly improved
				Process outcomes			

**Mistiaen 06 (telephone follow-ups) **[62]	Telephone follow-up (TFU) initiated by a hospital-based health professional (medical, nursing, social work, pharmaceutical) to a patient who is discharged to his/her own home setting (including a relative’s home).	33 RCT’s and controlled trials	hospital based healthcare professional in HIC	Compliance in cardiac surgery patients			1.68 [0.59, 4.78]
				Compliance in ED patients (making an appointment)			1.68 [0.59, 4.78]
				Compliance in ED patients (keeping an appointment)			1.58 [1.01, 2.48]
				Effect on knowledge in cardiac patients			1.44 [-0.25, 3.13]
				Effect on readmission(cardiac patients)			0.75 [0.41, 1.36]
				Effect on readmission in surgery patients			0.65 [0.28, 1.55]
				ED visits in surgery patients			1.47 [0.85, 2.53]

**Noordam 11 **[19]	The potential of mobile phones to improve maternal health services in Low and Middle Income Countries	Projects	LMIC	Accessing emergency obstetric care, improving the capacity of lesser trained health workers, empowering women		No	Narrative

**Shiffman 99 **[63]	Computer based guideline implementation	RCT: 9, NRCT: 01 time series: 10,	clinicians and other information providers in HIC	Guideline adherence		No	Improved in 14 of 18 studies
				Documentation			Improved in 4 of 4 studies.

**Tan 12 (telemedicine) **[64]	Telemedicine technology focused on education and support to the parents or caretakers of newborn infants receiving intensive care.	1 RCT	NICU staff in Indonesia		Length of hospital stay	Yes	-2.10 (-18.85-14.65)

Distance communication significantly improved immunization rates (Range: 6.4%-27.2%) and number of mammograms (Range: 14%-25%) [51] with non-conclsuive evidence on the use of telemedicine to support parents of high-risk newborns receiving intensive care [64]. Distance medicine technology also reported greater continuity of care by improving access and it should not be limited to physician-to-physician communication only [51]. Evidence also suggests that telephone consultation might reduce the number of surgery contacts and out-of-hours visits by general practitioners [53,54]. All the technological aspects of the interventions were reported to be well accepted by patients with some evidence of clinical benefits [56].

There is very limited evidence on interventions to promote information communication technologies (ICT), improvements in knowledge about the electronic sources of information and use of electronic databases and digital libraries by healthcare professionals [61,65]. Studies examining physician use of electronic records found mostly neutral or mildly positive effects on patient satisfaction (3.7%, 95% CI: 2.9-5.2%) [60] while computer based guideline implementation resulted in improved adherence [63]. No change in professional behavior was reported following electronic retrieval of health information.

## Discussion

At district level, audit and feedback mechanisms can effectively improve immunization rates; healthcare worker performance and compliance with desired practice; and prescription rates for generic drugs. Generalizability of these findings are however limited to HIC only. Involving local opinion leaders in informal/formal teaching, preceptor-ship and evidence based intervention dissemination can improve compliance with the desired practice. User-directed financial incentives have the potential to improve MNH outcomes, with CCT and maternal voucher schemes having the most significant positive impact across a range of outcomes. These findings are generalizable to both HIC and LMIC. There was limited and inconclusive evidence on the effectiveness of information technology with some positive impacts of distance communication on immunization rates and screening uptake. Evidence for structured interventions requiring electronic technologies are mainly evaluated in HIC settings hence limiting the generalizability of these findings to HIC only. This might be attributable to the gaps in access to and simultaneous underutilization of the existing electronic information resources in LMIC. Likewise, even within HIC, inequity exists in online information access between professionals in rural versus urban health settings.

There is a dearth of evidence from MNH perspectives in some domains of the district level inputs. Although financial incentives have been widely evaluated for their effectiveness in improving MNH outcomes; audits, feedbacks and information systems are mostly evaluated for general health outcomes. Furthermore, it is challenging to systematically measure and analyze data for subjective outcomes like patient/provider satisfaction and other process indicators. Reviews focusing on MNH specific interventions like maternal and perinatal mortality audits report lack of data to evaluate their effectiveness. There is also lack of qualitative data describing the individual components of the intervention for reproducibility since most of the interventions are not uniform but rather a range of approaches. For example, studies have not reported on the optimal format and frequency of audit and feedback [22]; supervision was also reported to be implemented in various ways with uncertain follow up periods [36,37].

Most of the district level interventions require a pre-existing primary health care service infrastructure and measures to ensure sustainability hence the major challenge is to ensure adequate political, financial, human and material commitments; optimal use of available resources; utilization of advanced technologies, changing management techniques including decentralization; measures to ensure accountability and effective community participation and intersectoral collaboration [66]. Hence it requires involvement of several stakeholders including policy makers, program managers and service providers from government organizations, private organizations, health development partners, technical assistance agencies, district directors and service providers. State leaders and key actors in the health sectors in LMIC along with the international community are proposed to translate the lessons learnt into actions and intensify efforts in order to achieve the goals set for MNH [67]. In LMIC, district health systems are still deprived of sustained policy attention and resources that it deserves, although more recently various forms of public private partnerships to improve MNH have emerged in LMIC whereby private organizations provide financial and technical support to refurbish and enhance the health services provided by the public sector but they are not formally evaluated for its impact.

Focus on basic primary health care interventions at the district level to improve coverage of effective public health interventions will help direct the attention towards essential preventive and promotive interventions and commodities required to deliver quality care to mothers and newborns [68]. Interventions like maternal and perinatal mortality audits and distance communication should be evaluated for effectiveness in improving MNH outcomes at the district level. Successfully implemented programs based on financial incentives to improve maternal and child health outcomes from Africa and Latin America can be simulated in other LMIC [12,50]. For these strategies to be more effective, it must be part of appropriate package of interventions, and technical capacity or support must be available. Programs integrating multiple interventions have shown maximum benefits on MNH outcomes as there is no single magic bullet intervention for reducing maternal and neonatal mortality [67]. These packages should then be monitored for possible unintended effects and evaluated using rigorous study designs to identify the best possible combination of the strategies tailored to the need of the district.

## Abbreviations

ANC: Antenatal care; AMSTAR: Assessment of Multiple Systematic Reviews; CCT: Conditional Cash Transfers; CI: Confidence Interval; CQI: Continuous Quality Improvement; ED: Emergency Department; FFS: Fee for Service; HIC: High Income Countries; ICT: Information Communication Technology; IMCI: Integrated Management of Childhood Illnesses; LMIC: Low Middle Income Countries; MD: Mean Difference; MNH: Maternal Newborn; NHI: National Health Insurance; PCP: Primary Care Physicians; PNC: Postnatal Care; RBF: Result Based Financing; RD: Rate Difference; RR: Relative Risk;

## Competing interests

We do not have any financial or non-financial competing interests for this review.

## Author contributions

All authors contributed to the process and writing of the manuscript.

## Peer review

Peer review reports are included in Additional file 1.

## Supplementary Material

Additional file 1 Click here for file

## Declarations

This article has been published as part of *Reproductive Health* Volume 11 Supplement 2, 2014: Quality of Care in Maternal and Child Health. The full contents of the supplement are available online at http://www.reproductive-health-journal.com/supplements/11/S2.
