# Supplementary material for: Evidence from district level inputs to improve quality of care for maternal and newborn health: interventions and findings
Source: Reprod Health. 2014 Sep 4;11(Suppl 2):S3. doi: 10.1186/1742-4755-11-S2-S3 (PMC4160920; doi:10.1186/1742-4755-11-S2-S3)
Supplement: Additional file 1 [file 1742-4755-11-S2-S3-S1.pdf]

Referee's comments to the authors– this sheet WILL be seen by the author(s) and published alongside the article

Article ref no.	
Title	Evidence from community level inputs to improve quality maternal and newborn health: Interventions and findings
Author(s)	Zohra S lassi; Jai K. Das; Rehana A Salam & Zulfiqar A Bhutta
Referee's name	J. Shea

When assessing the work, please consider the following points, where applicable:

[USE THE APPROPRIATE QUESTIONS FOR THE ARTICLE TYPE TO BE REVIEWED – SEE Reviewer Guidelines above]

1. Is the question posed by the authors new and well defined?
2. Are the methods appropriate and well described, and are sufficient details provided to replicate the work?
3. Are the data sound and well controlled?
4. Does the manuscript adhere to the relevant standards for reporting and data deposition?
5. Are the discussion and conclusions well balanced and adequately supported by the data?
6. Do the title and abstract accurately convey what has been found?
7. Is the writing acceptable?

Please make your report as constructive and detailed as possible in your comments so that authors have the opportunity to overcome any serious deficiencies that you find and please also divide your comments into the following categories:

- Major Compulsory Revisions (which the author must respond to before a decision on publication can be reached)
- Minor Essential Revisions (such as missing labels on figures, or the wrong use of a term, which the author can be trusted to correct)
- **Discretionary Revisions (which are recommendations for improvement but which the author can choose to ignore)**

Where possible please supply references to substantiate your comments.

When referring to the manuscript please provide specific page and paragraph citations where appropriate.

The quality of health care delivered at district level is an essential component of the overall quality of healthcare within the health system; thus the paper is relevant and important.

Abstract: The question/objective is not articulated.

Introduction: Although the Introduction has many key points, the writing and syntax undermine the integrity of the paper. The authors make good points in the opening paragraph, but the sentences are far too long. I would suggest that the language be simplified
What does this sentence mean: "District level healthcare is the zenith of primary health"?

.
The authors assume that all health systems are structured along similar lines, yet there is great variation between and also within countries. For example: "Programs such as the Safe Motherhood Initiative and the Integrated Management of Childhood Illness (IMCI) are based on district-level health systems ...". In some countries the IMCI strategy is located at Community Health Centres and Well-Baby Clinics i.e. at community level.
It is best to mention variations in district level healthcare up-front.

Leadership is not included in the definition of terms found in Panel 1.

District Level Characteristics: Each of the categories identified will benefit from revision so that the lengthy sentences are made more readable.
Governance and accountability: Governance is achieved, not delivered as stated in the manuscript.
Maternal and perinatal audits relate more to facility-based problem identification and problem solving.
Leadership and Supervision: This is an important aspect of healthcare and can be presented better. Supervision happens at levels with the healthcare organisation. Leadership was not discussed.
I am not sure what this means:" One of the emerging aspects of leadership and supervision interventions is the involvement of local opinion leaders to promote knowledge transfer of evidence into practice and ultimately improve health care[7]."
Financial Incentives: Good points in this section. I recommend that the authors separate the concepts in this sentence: "This involves provision of monetary benefits as a source of motivation for performing desired health related actions and not only aim to scale-up preventive health interventions but also provide free access to basic health care consequently creating a demand for health services"
It could possibly read: This involves the provision of monetary benefits as a source of motivation for performing desired health related actions. Interventions are aimed at creating a greater demand for health services and include the scale-up of preventive health interventions, as well as the provision of free access to basic health care.

Information system: Again, the opening sentence contains too much. The Health Information System is critical to improved service delivery and the quality of healthcare. It is advisable that the authors describe systems where paper-based HIS as well as technology-based HIS are used as there are many challenges in this area. In fact, technology-based systems have introduced an entire new set of challenges.

Search Strategy: The preliminary search strategy included all available systematic reviews on district level interventions as presented in the conceptual framework (presented in paper1). It seems that the initial search included a number of related but not relevant papers and these were excluded. The search strategy is generally well described, but I would advise that the authors be more specific about what keywords and what combination (strings) they searched for. It is important to note that Cochrane Systematic Reviews include only Randomised Controlled Trials (RCTs) and do not include a wide variety of other research designs, like mixed method studies. Social science and policy reviews may have been excluded from this review, and these are often excellent sources of quality of care research. General information about the study designs included in reviews other than Cochrane Systematic Reviews is recommended.

The diagram on page 28 is a good way of presenting the review process. I would recommend that some of the descriptions be expanded:
Titles screened – Initial title screening for appropriateness of topic
Abstracts screened – Abstracts evaluated against inclusion and exclusion criteria.
Full text screened – retrieval and review of full article against inclusion and exclusion criteria
All inclusion criteria met and no exclusion criteria

Findings: Definities for activities/processes central to QoC were not described in the findings nor in the Discussion. One example is: Involving local opinion leaders to promote evidence-based practice resulted in a 12% [RD: 12%, 95% CI: 6- 14.5%] absolute increase in compliance with the desired practice[30]" (p.6) – How were these opinion leaders engaged?
- For outcomes other than MNH, audit and feedback was found to increase the performance of health care workers and healthcare professionals' compliance with desired practice by 7% and4.3% respectively[15, 27]. (p.6) – What were these audit and feedback procedures?

Discussion: The discussion is generally good, although there are some shortcomings that need to be addressed. The purpose of the Discussion is to debate the evidence and identify the limitations of the review. The Discussion of the evidence can be improved.
- The Discussion is not systematically linked to the District Level Characteristics and Findings. For example, the first point in the Discussion relates to user directed financial incentives, while the first District Level Characteristic is about *Governance and accountability mechanisms.* The Discussion should flow out of the previous sections of the manuscript in a manner that facilitates a logical and coherent connectedness.
- On p.9 this sentence is inserted without any discussion related to the relevance of stakeholders: "District level inputs encompass diverse interventions involving several stakeholders including policy makers, program managers and service providers from government organizations, private organizations, health development partners, technical assistance agencies, district directors and service providers". Stakeholder engagement is an important aspect of quality of care and management processes; this point deserves some elaboration.
- There are sections in the Discussion that appear to be unrelated to the content of the manuscript. For example, this point emerges without any apparent link to the rest of the manuscript:" There is a need for a paradigm shift from disease-focused national priorities to basic primary health care interventions needed at the district level to improve coverage for effective public health interventions[59]." – (p. 9).

It is advisable that the authors address the issues that are not linked to the evidence in their review as limitations of the review.

In this manuscript there are several language/editing issues. For example:

- "Although these mechanisms have been used widely as a strategy to improve professional practice; these have not shown consistent effectiveness majorly due to the inconsistencies and variations involved in its implementation [2, 3]." – incorrect use of a semi-colon (p.3).
- Sentences are too long: "*:* Governance is delivered through a combination of strategies including clinical competence, patient involvement, risk management, use of information, staff management, maintaining medical registries, and implementation of continuous quality improvement (CQI) tools; while accountability involves audit

and feedback mechanisms that entail a systematic approach to ensure that the services are accountable for delivering quality healthcare.”

- Despite of being a vital ...” - grammatically incorrect
- There are several errors in this paragraph: We included seven [20, 28-33]reviews evaluating the impact of leadership and supervision with a median quality score of 8. The reviews focused on the impact of leadership and supervision for the primary health workers [28, 29]; involvement of local community leaders[30], nursing leadership [31, 33]and supervising counselors or psychotherapist [32]. Due to the wide range of reported outcomes, data could not be pooled for any outcome except for compliance in one review evaluating the impact of involving opinion leaders[30]. None of the reviews reported outcomes specific to MNH while other reported outcomes included compliance, patient satisfaction, provider’s practice and knowledge. The reviews were from both low- middle- income countries (LMIC) and HIC. The characteristics and findings of the included reviews are presented (p.6).
- “The reviews were from both low- middle- income countries (LMIC) and HIC” – low- and middle- (p.6)
-

The manuscript needs complete language/editing revision.

Referee's comments to the authors– this sheet WILL be seen by the author(s) and published alongside the article

Article ref no.	
Title	**Evidence from district level inputs to improve quality of maternal and newborn health: Interventions and Findings**<br><br>**Paper 3 of 5 paper-series review**
Author(s)	Rehana A Salam[1], Zohra S Lassi[1], Jai K Das[1] Zulfiqar A Bhutta[12]
Referee's name	Patji Alnæs-Katjavivi
Date for receipt of referee's comments	Monday 6th January 2014

When assessing the work, please consider the following points, where applicable:

[USE THE APPROPRIATE QUESTIONS FOR THE ARTICLE TYPE TO BE REVIEWED – SEE Reviewer Guidelines above]

8. Is the question posed by the authors new and well defined?
9. Are the methods appropriate and well described, and are sufficient details provided to replicate the work?
10. Are the data sound and well controlled?
11. Does the manuscript adhere to the relevant standards for reporting and data deposition?
12. Are the discussion and conclusions well balanced and adequately supported by the data?
13. Do the title and abstract accurately convey what has been found?
14. Is the writing acceptable?

Please make your report as constructive and detailed as possible in your comments so that authors have the opportunity to overcome any serious deficiencies that you find and please also divide your comments into the following categories:

- Major Compulsory Revisions (which the author must respond to before a decision on publication can be reached)
- Minor Essential Revisions (such as missing labels on figures, or the wrong use of a term, which the author can be trusted to correct)
- **Discretionary Revisions (which are recommendations for improvement but which the author can choose to ignore)**

Where possible please supply references to substantiate your comments.

When referring to the manuscript please provide specific page and paragraph citations where appropriate.

General comments:

This review of reviews is an impressive undertaking. I have enjoyed reading this paper in which I think the methodology is robust. It is challenging when attempting to summarize findings of interventions that differ in their setting geographically, culturally, and in availability of material and human resource. The reviews examine research that is both qualitative and quantitative. It is difficult to draw conclusions from such a very "mixed bag" of reviews that are applicable - and that can be expected to impact equally well in differing healthcare settings. Nonetheless, I believe that the strength of this paper is providing a comprehensive description of how "quality of care" in all its myriad forms and interpretations has been assessed up until the present day. Grouping the interventions as summarized on Panel 1, page 4, makes a useful tool for healthcare planners when trying to decide in which direction a given intervention will impact on the quality of health that is to be delivered.

I agree with the authors when they state "There is a need for a paradigm shift from disease-focussed national priorities to basic primary health care interventions…". The authors provide a template for how to approach health interventions in relation to quality of care, but there is a lack of argumentation/speculation as to why a given intervention has demonstrated itself to be useful /lacking impact.

The interventions and reviews that are examined are reported according to whether they work or whether they fail to demonstrate an impact. There is little examination as to the reasons why, in a given setting, an intervention has worked or not worked. These are the lessons that are perhaps most important to learn from. I found this aspect wanting in my reading. Perhaps if the <u>aim</u> of this paper was more clearly stated then this would not be an expectation I would have developed whilst reading. To give "user-directed financial schemes including maternal vouchers" as an example of a "successful" intervention – there is little discussion as to whether this impact is sustainable over time, and if so, what factors are important. Although there is "significant positive impact across a range of outcomes", this does not tell me whether mother&child morbidity and mortality are influenced adversely. My assumption is that improvement in antenatal/postnatal appointments will help reduce mortality and morbidity, but this assumption is not proved by the text in this paper.

The significance – whether clinical or statistical – of the interventions reviewed all depend on what sort of outcomes are believed to be important in relation to quality of care. The setting of maternity care I might hazard that universally one is interested in interventions that reduce mother&child mortality and morbidity, and that promote

mother&child satisfaction. Perhaps there could have been an attempt at addressing what is universally accepted as quality of care in maternity, and how the interventions reviewed impact directly – or indirectly.

Major compulsory revisions:

None, I have made my comments in other sections of this feedback form.

Minor essential revisions:

There are some minor typing errors in the text.

Discretionary revisions:

The aim of this paper should be stated.

The concept "Quality of care" is assumed. I have difficulty in appreciating what the authors mean when using this term. I believe that the reviews that they have assessed will probably differ in what they mean to be "quality of care", and yet this is not addressed in the discussion. I think that this is pertinent when considering that the reviews being compared concern clinical settings that differ greatly in terms of maternal and perinatal mortality and resources. The impact, or lack of impact, of the interventions and practices reviewed are not stated

Author responses

General comments:

This review of reviews is an impressive undertaking. I have enjoyed reading this paper in which I think the methodology is robust. It is challenging when attempting to summarize findings of interventions that differ in their setting geographically, culturally, and in availability of material and human resource. The reviews examine research that is both qualitative and quantitative. It is difficult to draw conclusions from such a very "mixed bag" of reviews that are applicable - and that can be expected to impact equally well in differing healthcare settings. Nonetheless, I believe that the strength of this paper is providing a comprehensive description of how "quality of care" in all its myriad forms and interpretations has been assessed up until the present day. Grouping the interventions as summarized on Panel 1, page 4, makes a useful tool for healthcare planners when trying to decide in which direction a given intervention will impact on the quality of health that is to be delivered.

I agree with the authors when they state "There is a need for a paradigm shift from disease-focussed national priorities to basic primary health care interventions…". The authors provide a template for how to approach health interventions in relation to quality of care, but there is a lack of argumentation/speculation as to why a given intervention has demonstrated itself to be useful /lacking impact. Thanks. We have now strengthened the findings and discussion section considerably to incorporate the suggestion.

The interventions and reviews that are examined are reported according to whether they work or whether they fail to demonstrate an impact. There is little examination as to the reasons why, in a given setting, an intervention has worked or not worked. These are the lessons that are perhaps most important to learn from. I found this aspect wanting in my reading. Perhaps if the aim of this paper was more clearly stated then this would not be an expectation I would have developed whilst reading. To give "user-directed financial schemes including maternal vouchers" as an example of a "successful" intervention – there is little discussion as to whether this impact is sustainable over time, and if so, what factors are important. Although there is "significant positive impact across a range of outcomes", this does not tell me whether mother&child morbidity and mortality are influenced adversely. My assumption is that improvement in antenatal/postnatal appointments will help reduce mortality and morbidity, but this assumption is not proved by the text in this paper. Thanks for pointing it out. We have tried to add in such details where possible.

The significance – whether clinical or statistical – of the interventions reviewed all depend on what sort of outcomes are believed to be important in relation to quality of care. The setting of maternity care I might hazard that universally one is interested in interventions that reduce mother&child mortality and morbidity, and that promote mother&child satisfaction. Perhaps there could have been an attempt at addressing what is universally accepted as quality of care in maternity, and how the interventions reviewed impact directly – or indirectly. Thanks. Agreed.

Major compulsory revisions:

None, I have made my comments in other sections of this feedback form.

Minor essential revisions:

There are some minor typing errors in the text.

Discretionary revisions:

The aim of this paper should be stated. **Added**

The concept "Quality of care" is assumed. I have difficulty in appreciating what the authors mean when using this term. I believe that the reviews that they have assessed will probably differ in what they mean to be "quality of care", and yet this is not addressed in the discussion. I think that this is pertinent when considering that the reviews being compared concern clinical settings that differ greatly in terms of maternal and perinatal mortality and resources. The impact, or lack of impact, of the interventions and practices reviewed are not stated in relation to.. **discussed in detail in paper 1 and 5 of the series**

In the background section, listing the factors beginning with a number will make for easier reading. For example, "They can be broadly divided into the four following categories, 1. Governance and accountability, …" **thanks. Modified**

Prof Jos van Roosmalen

Paper 3. Evidence from district level inputs to improve quality of maternal and newborn health: intervention and findings

The authors may respond to the following issues:

1. Abstract: while in the findings is written that supervision positively influenced provider's practice etc, and audit and feedback mechanisms were found to be associated with improved women and children's care, in the conclusion one reads: "Notwithstanding the benefits of financial incentives, audit, feedbacks and information systems should also be evaluated for their effectiveness etc." This is a not easily to understand conclusion after such findings. It also leads to extreme heterogeneity.Thanks. we have now corrected it.
2. Again: do not speak of "developing" and "developed" countries. We have corrected it.
3. A major problem of the whole study is that the review does not take "context" into account, while then evidence is presented from HIC and then from LIC or LMIC countries. It makes the "evidence" difficult to follow, because what may work in HIC does not necessarily work in other contexts. It would be of more help to have the different contexts better explained. Again I have doubts about the selection of papers as again I now feel one systematic review I was myself involved in, cannot be found somewhere: Nyamtema AS, et al. Maternal health interventions in resource limited countries: a systematic review of packages, impacts and factors for change. BMC Pregnancy Childbirth 2011; 11: 30 (also not in paper 4). I have doubts whether the search has been done in such a way, that all relevant papers have been found. Thanks and agreed. We have now added some context specific points in our discussion section to clarify. Regarding your review, we had it our initial screening

but we excluded this review as it evaluates the effectiveness of all interventions under review altogether and it was difficult for us to classify this paper under any one of our pre-defined domains in the conceptual framework (community, district and facility). It's a comprehensive review and thanks for bringing it up, we have now quoted some important points highlighted in this review and referenced it in our discussion.

4. On page 4, 4 of 5 references related to leadership and supervision are from 1976, 1984, 1988 and 1992, at least more than 20 years ago. Are there no more recent papers touchinh on leadership and supervision? we have now expanded this section and included some recent references
5. On page 5: what is "capitation"? Capitation is payment provided to healthcare professional on each patient enrolled. We have also clarified this in the text.
6. On page 8, last sentence before discussion: the use of electronic retrieval of health care information by health care providers to improve practice and patient care lacks the identification of context. In most LIC-settings such systems are not available for health care providers, sometimes they are available at the policy levels. Nobody will be surprised that the few available evidence stems from HIC. The relevance at LIC-level where most maternal and neonatal deaths are taking place is rather unclear. Agreed
7. On page 9: the statement that audits and feedbacks are mostly evaluated for general health outcomes is unclear as e.g. the audits I mentioned under paper 1 evaluate maternal and perinatal outcome.
8. On page 9, last par.: the statement "Interventions like maternal and perinatal mortality audits can be straightforwardly replicated …. and should be evaluated for effectiveness in improving MNH .." is a strange statements as you can find in the literature papers which report on the successes and failures of audit and feedback. Just look to the work of Van den Akker et al or Nyamtema et al. And I guess there will be many more. The statement by the authors suggest that these types of studies do not exist and still have to be performed, while you can easily find such studies. Agreed. We have now modified it.

The quality of health care delivered at district level is an essential component of the overall quality of healthcare within the health system; thus the paper is relevant and important.

Abstract: The question/objective is not articulated.

We have now clarified the objective

Introduction: Although the Introduction has many key points, the writing and syntax undermine the integrity of the paper. The authors make good points in the opening paragraph, but the sentences are far too long. I would suggest that the language be simplified

What does this sentence mean: “District level healthcare is the zenith of primary health”?

Thanks for pointing this out. We have now made the suggested changes in the text.

The authors assume that all health systems are structured along similar lines, yet there is great variation between and also within countries. For example: “Programs such as the Safe Motherhood Initiative and the Integrated Management of Childhood Illness (IMCI) are based on district-level health systems ...”. In some countries the IMCI strategy is located at Community Health Centres and Well-Baby Clinics i.e. at community level.

It is best to mention variations in district level healthcare up-front.

Thanks. We have now corrected it.

Leadership is not included in the definition of terms found in Panel 1.

Leadership and supervision added in panel 1

District Level Characteristics: Each of the categories identified will benefit from revision so that the lengthy sentences are made more readable.

Governance and accountability: Governance is achieved, not delivered as stated in the manuscript. Corrected

Maternal and perinatal audits relate more to facility-based problem identification and problem solving. Agreed

Leadership and Supervision: This is an important aspect of healthcare and can be presented better. Supervision happens at levels with the healthcare organisation. Leadership was not discussed. we have now modified the text and also added some text on leadership

I am not sure what this means:” One of the emerging aspects of leadership and supervision interventions is the involvement of local opinion leaders to promote knowledge transfer of evidence into practice and ultimately improve health care[7].” Clarified and corrected

Financial Incentives: Good points in this section. I recommend that the authors separate the concepts in this sentence: “This involves provision of monetary benefits as a source of motivation for performing desired health related actions and not only aim to scale-up preventive health interventions but also provide free access to basic health care consequently creating a demand for health services”

It could possibly read: This involves the provision of monetary benefits as a source of motivation for performing desired health related actions. Interventions are aimed at creating a greater demand for health services and include the scale-up of preventive health

interventions, as well as the provision of free access to basic health care. Thanks. The sentence is now replaced as suggested

Information system: Again, the opening sentence contains too much. The Health Information System is critical to improved service delivery and the quality of healthcare. It is advisable that the authors describe systems where paper-based HIS as well as technology-based HIS are used as there are many challenges in this area. In fact, technology-based systems have introduced an entire new set of challenges. Thanks. We have now considerably modified and expanded the text as suggested.

Search Strategy: The preliminary search strategy included all available systematic reviews on district level interventions as presented in the conceptual framework (presented in paper1). It seems that the initial search included a number of related but not relevant papers and these were excluded. The search strategy is generally well described, but I would advise that the authors be more specific about what keywords and what combination (strings) they searched for. It is important to note that Cochrane Systematic Reviews include only Randomised Controlled Trials (RCTs) and do not include a wide variety of other research designs, like mixed method studies. Social science and policy reviews may have been excluded from this review, and these are often excellent sources of quality of care research. General information about the study designs included in reviews other than Cochrane Systematic Reviews is recommended.

Thanks. We have now added the list of key and search term used. Information regarding the study designs included in reviews have been mentioned in the characteristics of included reviews table for ready reference.

The diagram on page 28 is a good way of presenting the review process. I would recommend that some of the descriptions be expanded:

Titles screened – Initial title screening for appropriateness of topic

Abstracts screened – Abstracts evaluated against inclusion and exclusion criteria.

Full text screened – retrieval and review of full article against inclusion and exclusion criteria

All inclusion criteria met and no exclusion criteria

We have now modified figure 1 as suggested.

Findings: Definities for activities/processes central to QoC were not described in the findings nor in the Discussion. One example is: Involving local opinion leaders to promote evidence-

based practice resulted in a 12% [RD: 12%, 95% CI: 6- 14.5%] absolute increase in compliance with the desired practice[30]" (p.6) – How were these opinion leaders engaged?

- For outcomes other than MNH, audit and feedback was found to increase the performance of health care workers and healthcare professionals' compliance with desired practice by 7% and4.3% respectively[15, 27]. (p.6) – What were these audit and feedback procedures?

Thanks. We have now added intervention description in the findings section, where available.

Discussion: The discussion is generally good, although there are some shortcomings that need to be addressed. The purpose of the Discussion is to debate the evidence and identify the limitations of the review. The Discussion of the evidence can be improved.

- The Discussion is not systematically linked to the District Level Characteristics and Findings. For example, the first point in the Discussion relates to user directed financial incentives, while the first District Level Characteristic is about *Governance and accountability mechanisms.* The Discussion should flow out of the previous sections of the manuscript in a manner that facilitates a logical and coherent connectedness.

Thanks for pointing that out. We have now followed the discussion in the same manner as the findings.

- On p.9 this sentence is inserted without any discussion related to the relevance of stakeholders: "District level inputs encompass diverse interventions involving several stakeholders including policy makers, program managers and service providers from government organizations, private organizations, health development partners, technical assistance agencies, district directors and service providers". Stakeholder engagement is an important aspect of quality of care and management processes; this point deserves some elaboration. We have now modified the discussion

- There are sections in the Discussion that appear to be unrelated to the content of the manuscript. For example, this point emerges without any apparent link to the rest of the manuscript:" There is a need for a paradigm shift from disease-focused national priorities to basic primary health care interventions needed at the district level to improve coverage for effective public health interventions[59]." – (p. 9). We have now modified the discussion as suggested.

It is advisable that the authors address the issues that are not linked to the evidence in their review as limitations of the review. Thanks for pointing this out.

In this manuscript there are several language/editing issues. For example:

- "Although these mechanisms have been used widely as a strategy to improve professional practice; these have not shown consistent effectiveness majorly due to the inconsistencies and variations involved in its implementation [2, 3]." – incorrect use of a semi-colon (p.3).Corrected
- Sentences are too long: "*:* Governance is delivered through a combination of strategies including clinical competence, patient involvement, risk management, use of information, staff management, maintaining medical registries, and implementation of continuous quality improvement (CQI) tools; while accountability involves audit and feedback mechanisms that entail a systematic approach to ensure that the services are accountable for delivering quality healthcare." Corrected
- Despite of being a vital ..." - grammatically incorrect Corrected
- There are several errors in this paragraph: We included seven [20, 28-33]reviews evaluating the impact of leadership and supervision with a median quality score of 8. The reviews focused on the impact of leadership and supervision for the primary health workers [28, 29]; involvement of local community leaders[30], nursing leadership [31, 33]and supervising counselors or psychotherapist [32]. Due to the wide range of reported outcomes, data could not be pooled for any outcome except for compliance in one review evaluating the impact of involving opinion leaders[30]. None of the reviews reported outcomes specific to MNH while other reported outcomes included compliance, patient satisfaction, provider's practice and knowledge. The reviews were from both low- middle- income countries (LMIC) and HIC. The characteristics and findings of the included reviews are presented (p.6). We have now modified the text and language
- "The reviews were from both low- middle- income countries (LMIC) and HIC" – low- and middle- (p.6) Corrected
-
